# The WE-Study: does botulinum toxin A make walking easier in children with cerebral palsy?: Study protocol for a randomized controlled trial

**DOI:** 10.1186/s13063-016-1772-8

**Published:** 2017-02-06

**Authors:** Siri Merete Brændvik, Karin Roeleveld, Guro Lillemoen Andersen, Anne Elisabeth Ross Raftemo, Kjersti Ramstad, Jasmina Majkic-Tajsic, Torarin Lamvik, Bendik Lund, Turid Follestad, Torstein Vik

**Affiliations:** 1 Department of Neuromedicine and Movement Science, Norwegian University of Science and Technology, NTNU, Trondheim, Norway; 2 Clinical Services, St. Olav’s University Hospital, Trondheim, Norway; 3 Children’s Center, Vestfold Hospital Trust, Tønsberg, Norway; 4 Department of Laboratory Medicine, Children’s and Women’s Health, Norwegian University of Science and Technology, NTNU, Trondheim, Norway; 5 Department of Clinical Neurosciences for children, Oslo University Hospital, Oslo, Norway; 6 Division of Child and Adolescents Health, University Hospital of North Norway, Tromsø, Norway; 7 Department of Orthopedics, St. Olav’s University Hospital, Trondheim, Norway; 8 Department of Pediatrics, St. Olav’s University Hospital, Trondheim, Norway; 9 Department of Public Health and General Practice, Norwegian University of Science and Technology, NTNU, Trondheim, Norway

**Keywords:** Cerebral palsy, Spasticity, Botulinum toxin A, Walking, Energy cost, Activity, Pain

## Abstract

**Background:**

Intramuscular injections of botulinum toxin A (BoNT-A) have been a cornerstone in the treatment of spasticity for the last 20 years. In Norway, the treatment is now offered to two out of three children with spastic cerebral palsy (CP). However, despite its common use, the evidence for its functional effects is limited and inconclusive. The objective of this study is to determine whether BoNT-A makes walking easier in children with CP. We hypothesize that injections with BoNT-A in the calf muscles will reduce energy cost during walking, improve walking capacity, increase habitual physical activity, reduce pain and improve self-perceived performance and satisfaction.

**Methods/design:**

This randomized, double-blinded, placebo-controlled, multicenter trial is conducted in a clinical setting involving three health regions in Norway. Ninety-six children with spastic CP, referred for single-level injections with BoNT-A in the calf muscles, will be invited to participate. Those who are enrolled will be randomized to receive either injections with BoNT-A (Botox®) or 0.9% saline in the calf muscles. Stratification according to age and study center will be made. The allocation ratio will be 1:1. Main inclusion criteria are (1) age 4 − 17.5 years, (2) Gross Motor Function Classification System levels I and II, (3) no BoNT-A injections in the lower limbs during the past 6 months and (4) no orthopedic surgery to the lower limbs during the past 2 years. The outcome measures will be made at baseline and 4, 12 (primary endpoint) and 24 weeks after injections. Primary outcome is change in energy cost during walking. Secondary outcomes are change in walking capacity, change in activity, perceived change in performance and satisfaction in mobility tasks, and pain. The primary analysis will use a linear mixed model to test for difference in change in the outcome measures between the groups. The study is approved by the Regional Ethical Committee and The Norwegian Medicines Agency. Recruitment started in September 2015.

**Discussion:**

The evaluation of effect is comprehensive and includes objective standardized tests and measures on both impairment and activity level. Results are to be expected by spring 2019.

**Trial registration:**

ClinicalTrials.gov, NCT02546999. Registered on 9 September 2015.

**Electronic supplementary material:**

The online version of this article (doi:10.1186/s13063-016-1772-8) contains supplementary material, which is available to authorized users.

## Background

Cerebral palsy (CP) is the most common cause of neurological disability in childhood, and the motor signs associated with the condition include primary neuromuscular deficits, such as spasticity, muscle weakness, and decreased selective motor control, and secondary musculoskeletal problems such as bony malformations and contractures [1].

Impaired walking is a main clinical feature of CP, and although approximately 70% of children with CP are able to walk, they all experience varying degrees of restrictions related to this function [2]. Such restrictions include reduced walking speed [3], impaired balance [4] and high energy costs (ECs) during walking [5]. The ECs during walking increase with increasing level of disability [6] and age [7], and these impairments may, therefore, in part contribute to fatigue; a common complaint in children and youths with CP [8]. Moreover, recurrent musculoskeletal pain is common, affecting both general activity and walking [9, 10]. As a consequence, children with CP are in general less active than their peers, and they less often take part in social activities [11].

The dominating motor disorder in CP is spasticity, defined as a velocity-dependent increase in resistance to passive movement [12], present in more than 80% of the children [2]. The introduction of intramuscular injections of botulinum toxin A (BoNT-A) in the 1990s represented a significant breakthrough in the treatment of spasticity. BoNT-A is a neuromuscular paralyzing agent and it results in a dose-dependent chemo-denervation of the muscle by inhibiting the release of the neurotransmitter acetylcholine at the neuromuscular junction. Currently, treatment with BoNT-A is offered to as many as two thirds of all children with spastic CP and more than half of the children are able to walk independently [13].

Among children able to walk, a main goal of this treatment is to improve gait [14], and thereby improving the potential for higher levels of activity and participation. It is well-documented that BoNT-A reduces spasticity [15, 16]; however, despite being commonly used, evidence for functional effects is limited and inconclusive [17, 18]. In a systematic review [19], including eight randomized control trials (RCTs) the effects on walking were specifically addressed. The authors found that five of the eight studies were of low methodological quality, and seven got low scores on clinical relevance. The main limitations of the included studies related to differences in outcome measures, lack of a-priori defined effect sizes, and variations in follow-up time. The authors of the systematic review, therefore, had to rely on their “general impression” of improvement in “any functional outcome.” Based on this “general impression” the authors concluded that the use of BoNT-A with usual care or physiotherapy seemed to improve walking of children with CP emphasizing that their conclusions should be appraised carefully owing to the limited quality of included trials. Other long-term follow-up studies, taking a pragmatic approach in order to reflect clinical practice, were unable to document cumulative or persistent benefit on walking of repeated BoNT-A injections [20]. Thus, a fair summary of the existing medical literature is that the evidence for an effect of BoNT-A injections in the leg muscles on walking is limited and inconclusive.

The main objective of the current trial is, therefore, to investigate whether injections of BoNT-A in the calf muscles (m. gastrocnemius and m. soleus) make walking easier in children and adolescents with CP within a time span of 6 months, as judged by reduced EC during walking. Secondary objectives are to explore whether injections of BoNT-A increase activity, improve walking capacity, decrease musculoskeletal pain, and improve perceived performance and satisfaction related to mobility tasks.

We hypothesize that injections of BoNT-A in the calf muscles will reduce EC during walking, improve walking capacity, increase habitual physical activity and reduce pain. Moreover, we hypothesize that injections with BoNT-A will improve perceived performance and satisfaction related to ambulation. Secondary, we hypothesize that younger children and children with lower gross motor function will improve more in EC than older children and children with greater gross motor function.

In a substudy of this RCT, we aim to identify different CP phenotypes of responders to BoNT-A treatment and, moreover, to gain insight into the processes underlying BoNT-A-induced changes in walking. On that account, we want to identify gait and muscle activation patterns, spasticity and strength characteristics related to the response of BoNT-A on our primary and secondary outcome measures. Moreover, semistructured qualitative interviews will be conducted on a subset of participants (minimum 25) and their parents. Details of these studies are not presented in this paper.

## Methods/design

### Study design and setting

The WE-Study is designed as an industry-independent, double-blinded, placebo-controlled, superiority trial where participants are randomized to receive either injections of BoNT-A or saline (placebo) in the calf muscles. The allocation ratio will be 1:1. It is a multicenter study conducted in a clinical setting, involving three out of four health regions in Norway as well as the Norwegian University of Science and Technology, NTNU. The study will be conducted according to the Declaration of Helsinki and ICH-Good Clinical Practice guidelines. The study protocol follows the SPIRIT 2013 (Standard Protocol Items: Recommendations for Interventional Trials) guidelines [21]. The SPIRIT Checklist (Additional file 1) and figure (Table 1) are provided.

**Table 1:** Standard Protocol Items: Recommendations for Interventional Trials (SPIRIT) figure

Study period						
	Enrolment	Allocation	Post allocation			Close-out
Time point	Baseline		Intervention	P1 4 weeks post	P2 12 weeks post	P3 24 weeks post
Enrollment:						
Eligibility screen	x					
Informed consent	x					
Allocation		x				
Interventions:						
Saline			x			
Botulinum toxin A			x			
Assessments:						
Energy cost	x			x	x	x
Habitual physical activity	x			x	x	x
Pain	x			x	x	x
Perceived change in performance and satisfaction	x			x	x	x
Spasticity test	x			x	x	x
Passive range of motion in the ankle	x			x	x	x

Monitoring will be carried out according to a predefined plan, independent of the sponsor and with no competing interests. Protocol modifications will be reviewed by the Regional Ethics Committee (REC). Substantial modifications will also be reviewed by The Norwegian Medicines Agency. Processing of personal study data will be done according to procedures approved by the data protection official at each study center. The trial registration data are provided in Table 2.

**Table 2:** Trial registration data

Data category	Information
Primary registry	ClinicalTrials.gov NCT02546999
	EudraCT Number 2014-002539-32
Secondary identifying numbers	REC North 2013/1195
	The Norwegian Medicines Agency 14/15799-9
Sponsor	St. Olav’s University Hospital, Trondheim, Norway
Contact	Siri.merete.brendvik@stolav.no
Short title	The WE-Study (Walking Easier with cerebral palsy)
Scientific title	Does botulinum toxin A make walking easier in children with cerebral palsy?
Country of recruitment	Norway
Population	Children with cerebral palsy (4 − 17.5 years)
Intervention	Active comparator: botulinum toxin A (Botox®)
	Placebo comparator: saline
Study type	Interventional parallel-group, randomized, double-blinded
	Phase IV
	Primary purpose: treatment effect
First enrollment	September 2015
Target sample size	96
Primary outcome	Energy cost during walking
Secondary outcome	Activity, pain, walking capacity, perceived benefit

### Participants

Eligible to participate are children and adolescents between 4 and 17.5 years of age (at the time of inclusion) with spastic unilateral or bilateral CP. The gross motor function of the participants should be at levels I or II according to the Gross Motor Function Classification System (GMFCS) [22] and they should not have been treated with BoNT-A in the lower limbs during the past 6 months or have undergone orthopedic surgery to the lower limbs in the last 2 years prior to study participation. Moreover, participants must be able to take verbal instructions. Exclusions are made based on the following criteria: history of prior adverse reactions to BoNT-A, presence of infection at the proposed injection site(s), subclinical or clinical evidence of defective neuromuscular transmission or other underlying neurological disorders that may be affected by BoNT-A injections, use of aminoglycoside antibiotics or spectinomycin, or other medicinal products that interfere with neuromuscular transmission (e.g., neuromuscular blocking agents), women with childbearing potential not using contraception, pregnant or breastfeeding women, children needing deep sedation, or any other reason in the opinion of the investigator. Children who receive concurrent injections in the upper limbs may be included in the study. Each study center will have one person in the study team responsible for introducing eligible participants to the study and the information given will be both orally and written. The same person will be responsible for obtaining written consent before baseline testing.

Once enrolled in the study, every reasonable effort will be made to avoid dropout and there will be close communication with participants and caregivers in the follow-up period. Patients may be discontinued from study treatment and assessments at any time. Specific reasons for discontinuing a patient for this study are: voluntary discontinuation by the patient, safety reason as judged by the principal investigator, major protocol deviation, incorrect enrollment, i.e., the patient does not meet the required inclusion/exclusion criteria for the study, deterioration in the patient’s condition which, in the opinion of the principal investigator, warrants study discontinuation (to be recorded as an adverse event; AE) and patient noncompliance to study treatment and/or procedures. Reason for discontinuation will be recorded in the web-based Case Report Form (eCRF) used in the study, and the participant will be offered an assessment including both primary and secondary outcomes before leaving the trial. After completing the study period, the participants will continue their regular follow-up at the hospital. Insurance coverage for this study is made through membership of the Drug Liability Association.

### Randomization and blinding

Eligible participants who have provided informed consent, either on behalf of themselves, by proxy (parents) or both, are randomized to either injections of BoNT-A or saline. The randomization will be performed using prerandomized lists at the four study centers, made by the unit of Applied Clinical Research at the Norwegian University of Science and Technology, using a web-based randomization system, including stratification for age (4–10 years and 11–17.5 years) and study center. The randomization lists are distributed to, and stored at, each respective study site’s pharmacy department which allocates the participants to either BoNT-A injections or saline (allocation ratio 1:1). The appropriate solution of BoNT-A or saline will then be prepared at the pharmacy and delivered in syringes to the researchers. In a pilot, we found that the two solutions were indistinguishable regarding smell and appearance. Accordingly, trial participants and their caregivers, and all other persons involved in treatment and outcome assessment are blinded to group allocation. The procedure for emergency unblinding is included in the study’s Trial Master File (TMF) and will be carried out by the responsible pharmacy in cooperation with coordinating national investigator of the study.

### Treatment procedure

The dosage of BoNT-A (Botox®; Allergan) used in this study is based on one systematic review [23], one original paper [24] and two international expert consensus papers [25, 26]. The total maximum body dose of Botox® will be 420 Units. Maximum dose per injection site will be 50 Units. The gastrocnemius muscle will receive 5–6 Units Botox® per kilogram, with a maximum of 180 Units in each leg. The soleus muscle will receive 2 Units Botox® per kilogram with a maximum dosage of 60 Units in each leg. The maximum injected volume per injection site will be 0.5 ml of a solution of 100 Units of Botox® in 1 ml 0.9% saline, or 0.9% saline only (placebo). The placebo dose will be the same dose in milliliters as the reconstituted Botox®. The injections are given using ultrasound-guidance, allowing quick visual identification of the target muscle and exact localization of the needle in the desired position. The participants will receive the treatment using local anesthesia and conscious sedation with oral or nasal benzodiazepines if desired by the participants and their caregivers. The sedation procedures will be given in accordance with standard guidelines used in each of the participating centers.

### Study visits

**Fig. 1:**
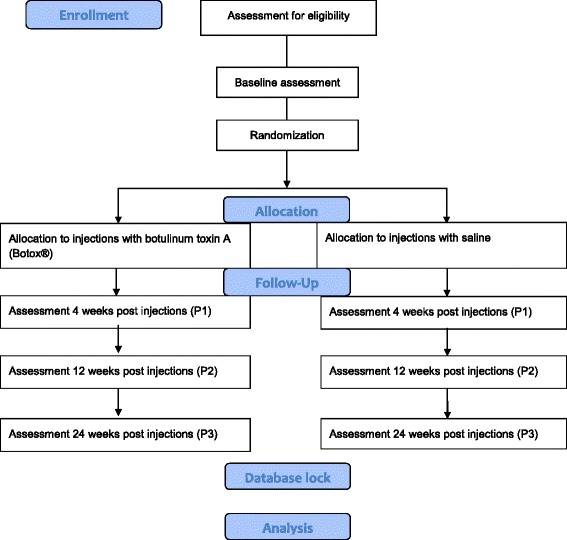
Flow of participants through study. Figure 1 shows the flow of participants through the study according to the Consolidated Standards of Reporting Trials (CONSORT) guidelines

Flow of participants and the timeline is presented in Fig. 1. The primary endpoint will be 12 weeks post injection, when a clinical effect can be expected. Outcomes at 4 and 24 weeks post injection will also be evaluated in order to control for the specific paralyzing effect on the muscle [27] and a possible delayed functional effect [15], respectively. Thus, the outcome measures will be assessed at baseline and 4, 12 and 24 weeks after injections (Fig. 1: P1, P2 and P3).

At each visit, there will be a face-to-face session with the child and the parents to map the period from the last visit. In addition, any AE will be recorded in the eCRF, and action taken according to procedure included in the TMF. All changes in concomitant medication (including vitamins, herbal preparation and other “over-the-counter” drugs) used by the patient will also be recorded in eCRF. Moreover, the content and intensity of supportive therapy, such as specific exercise programs, physiotherapy and ankle-foot orthoses (AFO) will be recorded in detail. Since such supportive therapy is not standardized in Norway and, therefore, will vary between centers according to their clinical guidelines, stratification by center at randomization is made.

At baseline, the following subject characteristics are reported: GMFCS level, CP distribution (uni/bilateral) number of previous BoNT-A treatments in calf muscles, previous surgeries to the lower limbs, age and gender. At each visit, spasticity is evaluated using the clinical Tardieu Test [28], and weight and height are also registered.

### Primary outcome measure

The primary outcome measure is energy cost (EC) in J/kg/m during walking, obtained from a 5-Minutes Walk Test (5MWT) at a self-chosen comfortable speed [29] with simultaneous gas-exchange measurements [3]. It has been reported that children with CP have an EC during walking corresponding to 6.84 J/kg/m (standard deviation (SD) 2.0 J/kg/m) and that the smallest detectable difference of this measure is 0.464 J/kg/m (or 6.8%) [29]. Taking this information into consideration we defined a 10% improvement in the intervention group (i.e., 0.684 J/kg/m) to be clinically significant. For children using an AFO on a regular basis, this assessment will be carried out both with and without the orthosis.

### Secondary outcome measures

Secondary outcomes are:

1. Walking capacity, reflected as (a) distance walked during a 1-Minute Walk Test (1MWT) where the participant is walking as fast as possible without running [30] and (b) perceived exertion during walking assessed by the use of the OMNI Rating of Perceived Exertion scale (OMNI-RPE), measured after the 5MWT at comfortable speed and after the 1MWT [31]. In the OMNI-RPE the children are asked to rate their perceived exertion on a 10-point scale (0–10) by the use of a series of four pictures of a child walking up a hill becoming progressively more and more tired. It is validated for children with CP [31]
2. Habitual physical activity, measured by two body-worn accelerometers (Axivity Ltd.); one at the thigh and one at the lower back, over a period of seven consecutive days following visits at baseline, and at P1, P2 and P3. The accelerometer as a measure of habitual physical activity is reported to be feasible in children with CP [32] and valid in reflecting time in sitting, lying, standing and walking [33]. In our study, two accelerometers are used in order to differentiate between sitting and lying
3. Perceived change in performance and satisfaction, measured by The Canadian Occupational Performance Measure (COPM). The COPM is used to detect changes in self-perceived activity performance in the areas of self-care, productivity (i.e., school) and leisure. At baseline, the instrument will be administered as a semistructured interview that focuses on activities that the participant wants, needs or is expected, to perform. Through the interview, the child will be allowed to identify and prioritize up to three “problems” related to gross motor function. Performance and satisfaction related to each problem are then scored on a 10-point ordinal scale (1–10), where a higher score reflects greater performance and satisfaction, respectively. Rescoring will be made at P1, P2 and P3. The COPM in its original version, as well as in the Norwegian translation, has been found to be a valid, reliable and responsive outcome measure ranging from satisfactory to excellent [34]
4. Pain during the last 2 weeks will be assessed stepwise. Participants will be asked to record all pain sites on the body outline from the Brief Pain Inventory (BPI), the Norwegian version [35] and to respond to the two questions on pain (“how much” and “how often”) from the Child Health Questionnaire (CHQ, Norwegian version) [36]. To capture the level of pain interference with function, pain interference with the three BPI items general activity, walking and sleep will be recorded on 0 (no interference) to 10 (complete interference) rating scales. In cases of calf pain, identical recordings will be made for the right and left leg separately and the child will be asked to indicate maximum calf pain intensity on the Faces Pain Scale-Revised (FPS-R) [37]. To secure the view from both parents and the young people with CP separately, parents will respond to a questionnaire while their child is occupied with test procedures, and the young people with CP will give their responses in an interview.

In addition, at all four visits, spasticity in the calf muscles will be assessed by the clinical Tardieu Test [28] and passive range of motion in the ankle joint will be measured by a manual goniometer. Moreover, during the 5MWT and the 1MWT, concurrent heart rate monitoring and accelerometry will be obtained. All data collection will be carried out by trained assessors and pilots on all outcomes were made before trial start. Collected data will be entered on site into each participant’s eCRF by the assessors and the data will be checked by the data manager in the study after each entry.

### Sample size and power

Sample size calculations were performed for a two-sample (two-sided) *t* test for comparing the change in the primary outcome measure, EC, from baseline to 12 weeks post intervention between the two groups. The estimate was based upon a mean difference in change of 0.684 6.84 J/kg/m [29]. The SD of change was set at 1.0 based on other intervention studies [3, 38], in which the SD of change is found to be approximately half of the baseline SD [29], and assuming a baseline SD of 2.0 J/kg/m [29]. Thus, to detect a clinical significant improvement in the primary outcome, measure, reflected as a 10% decrease in EC (from 6.84 J/kg/m), with a power of 80%, using a two-sided *t* test and a 5% significance level, a sample size of 32 children/adolescents per group is necessary. Allowing for a dropout of 30%, 48 children/adolescents are needed. In practice, the test will be performed as a post-hoc test for a linear mixed model (LMM), but the sample size calculation for the *t* test for the change from baseline to post is assumed to be conservative in that respect.

### Statistical analysis

The statistical analysis for the primary aim will be performed using a LMM including data from baseline and 4, 12 and 24 weeks post intervention. The test for difference in change in the primary outcome measure from baseline to 12 weeks between the treated and placebo group will be done using a post-hoc test following the LMM. Age, GMFCS level, number of prior BoNT-A treatments and study center will be considered as potential covariates. A similar model will also be used to study the secondary outcome measures. The LMM handles missing data for the outcome measure. Nonparametric tests will be used to test for a between-group difference in change score for secondary outcome measures expressed with ordinal data, and for scalar data in case the requirements for the parametric tests are not met. The effect size will be expressed as mean difference between the groups in change from baseline to 12 weeks with a 95% confidence interval.

In addition to investigating the independent effect of BoNT-A on walking, the associations between the change scores of the primary and secondary outcome measures will also be studied.

The level of significance is set to 0.05 and no formal adjustments for multiple testing will be carried out.

A complete plan for the analyses of the outcome measures will be made before unmasking group adherence. Results will be reported according to the Consolidated Standards of Reporting Trials (CONSORT) Statement [39].

## Discussion

Despite the common use of BoNT-A, the evidence for beneficial functional effects on walking in children with spastic CP is insufficient and there is, therefore, a strong need for high-quality RCTs that can fill this knowledge gap.

The ethical concerns in this study are related to the injections of placebo (saline) into the leg muscles of children in the control group. Such injections can be as painful as BoNT-A injections despite the use of local anesthesia and mild sedation. Deep sedation can usually be given to anxious children or to children who experience pain during the treatment procedure. However, the Ethical Committee did not approve the use of deep sedation to children in the control group. Since most patients are treated without deep sedation, this restriction is not expected to affect the number of participants substantially. In addition, the regular treatment of children in the control group will be delayed by at least 6 months. With respect to the latter, however, a recently published paper by Hastings-Ison and co-workers [40]. comparing 4 versus 12 months’ treatment frequency of BoNT-A injections, recently reported no significant difference in passive ankle dorsiflexion. Thus, the results of that study, as well as our clinical experience, and in the perspective of the total BoNT-A treatment of these children, this postponement is unlikely to affect the long-term outcome.

Moreover, it may be considered an ethical issue that more than half of the children with GMFCS levels I and II are treated with this drug, and a proportion of them require deep sedation during treatment if the drug does not have the assumed effects. Therefore, it seems to us that the use of placebo in this study is in line with general requirements for better evidence of pediatric treatment, including high-quality RCTs [41].

This current trial has several strengths. The study is comprehensive, including a relatively homogenous population of children classified with GMFCS levels I and II. Moreover, the intervention comprises single-level injections in the calf muscles only. Including participants with multilevel injections as well would challenge the interpretation of the results. Finally, the evaluation of effect is comprehensive and includes objective standardized tests and measures on both the impairment and activity level according to the ICF [42]. Effect on participation is not evaluated explicitly, since we consider that the follow-up time of 24 weeks will be too short to expect significant clinical changes in this dimension. Nonetheless, the COPM may give some indications of possible effects on participation.

The randomization procedure is stratified according to age and study center. The age range of the participants is quite wide, from 4 years to 17.5 years. Since there is possibly an effect of age, due to assumed structural and functional changes in the muscle as a result of growth and spasticity [43], two age groups are created (4–9 and 10–17.5 years) to make sure that age is evenly distributed among BoNT-A and placebo groups. The differences between the study sites regarding supportive therapy will be controlled through the stratification of the randomization. Moreover, the detailed description of this therapy applied to each participant will enable us to correct for potential differences in supportive therapy.

A main challenge in the project is recruitment of participants; the patient base is small, and the possibility of receiving placebo may cause some reluctance to participate. In case of slow recruitment, we plan to implement one more study site in a fourth health region in Norway.

### Trial status

This trial started recruiting participants medio September 2015 and recruitment period is estimated to be 3 years. Results are, therefore, to be expected during spring 2019. All personnel who have contributed significantly with the planning and performance of the study (Vancouver convention 1988) may be included in the list of authors.

In addition, to be submitted for publications in peer-reviewed journals and communicated to the participants, the results of this study will also be submitted to the Competent Authority and the Ethics Committee according to EU and national regulations.

## Additional file

Additional file 1: SPIRIT Checklist. (PDF 129 kb)

## Abbreviations

1MWT: 1-Minute Walk Test
5MWT: 5-Minutes Walk Test
AE: Adverse event
BoNT-A: Botulinum toxin A (Botox®)
CP: Cerebral palsy
EC: Energy cost
eCRF: Web-based Case Report Form
GMFCS: Gross Motor Function Classification System
OMNI-RPE: OMNI Rating of Perceived Exertion scale
TMF: Trial Master File
